# Reflexive Fusional Vergence and Its Plasticity Are Impaired in Convergence Insufficiency

**DOI:** 10.1167/iovs.61.10.21

**Published:** 2020-08-11

**Authors:** Ian M. Erkelens, William R. Bobier

**Affiliations:** School of Optometry and Vision Science, University of Waterloo, Waterloo, Ontario, Canada

**Keywords:** convergence, adaptation, convergence insufficiency, vergence plasticity, neural mechanisms

## Abstract

**Purpose:**

We compared the adaptive capacities of reflexive fusional convergence and divergence in 10 participants with untreated convergence insufficiency (CI) to 10 age-matched binocularly normal controls (BNCs) in an effort to elucidate the functional basis of CI.

**Methods:**

Vergence responses were monitored binocularly at 250 Hz using video-based infrared oculography, while single and double-step disparity stimuli were viewed dichoptically. The double-step stimuli were designed to induce an adaptive increase in the convergence or divergence reflexive fusional response dynamics.

**Results:**

As expected, convergence responses in the CI population were significantly slower at baseline (BNC 12.0 ± 1.8°/s vs. CI 7.4 ± 2.5°/s; *P* < 0.001), but divergence response velocities were similar between groups (*P* = 0.38). Critically, we observed an impaired adaptive change in convergence peak velocities in the CI group when compared to BNCs (–18.2% ± 27.3% vs. 25.4% ± 9.8%; *P* < 0.001). Adaptive changes in reflexive fusional divergence responses were similar between groups (*P* > 0.5) and significantly less robust when compared to BNC convergence.

**Conclusions:**

The results support the hypothesis that the adaptive capacities of vergence are related to the strength of the underlying reflexive fusional response. Combined, the evidence suggests that the clinical condition of convergence insufficiency is underpinned by an underdeveloped or perturbated reflexive fusional vergence response mechanism. We relate these observations to different clinical guidelines for the management and treatment of this condition.

Vergence eye movements align the two visual axes and provide the mechanism for maintaining single binocular vision.^1^ Convergence insufficiency (CI) is the most common non-strabismic oculomotor dysfunction.^2^^,^^3^ Individuals with CI struggle to maintain binocular fusion at near.^4^^,^^5^ CI manifests as symptoms of visual fatigue, headaches, and blurred vision during periods of near work, such as reading.^4^^,^^6^ In more severe cases of CI, patients report intermittent or constant diplopia when attempting to fixate at near. CI has traditionally been recognized as developmental in nature; however, recent investigations have reported CI-like signs and symptoms in a significant number of individuals suffering traumatic brain injuries.^7^^,^^8^ The diagnosis of CI in adolescences has been associated with deficiencies in reading performance,^9^ impairments of visual attention, and behavioral deficits.^10^^–^^13^ There is also evidence that adults with CI perform worse on tests of cortical integrative functions when compared to age-matched controls.^14^ The impact of CI on these and other cognitive processes, as well as the most appropriate rehabilitative therapies for CI, remain the source of much debate,^15^^–^^18^ in part due to a limited understanding of the mechanisms that underlie it. In addition, other oculomotor deficits such as accommodative insufficiency are common comorbidities and can exacerbate symptoms.^2^^,^^8^^,^^19^^–^^21^

Step changes in retinal disparity generate a reflexive inward (convergence) or outward (divergence) rotation of the eyes in order to “fuse” the disparate images via alignment of the two visual axes. Like saccades, fusional vergence is characterized by a pulse–step of neural innervation.^22^^–^^26^ The pulse component generates a reflexive, preprogrammed response, and the step component integrates this preprogrammed response with visual feedback, guiding the eyes to the new desired vergence angle and holding them there.^22^ One of the hallmark laboratory signs of CI is reduced fusional convergence response amplitudes and peak velocities to step changes in disparity.^7^^,^^27^^–^^30^ In traumatically acquired CI, these convergence responses fatigue quickly, showing poor endurance.^31^ This is in addition to the classic clinical presentation of a reduced near point of convergence (NPC), large dissociated exophoria at near and reduced positive relative vergence.^2^^,^^32^^,^^33^ Relative fusional vergence ranges are a clinical metric of the underlying plasticity of tonic vergence innervation.^34^^–^^36^ We have demonstrated in the laboratory that the adaptive uncoupling of this convergence-driven accommodation response is reduced in patients with CI when viewing at near distances^37^ and is associated with the visual fatigue and defocus and/or diplopia symptoms found in CI.^38^ Neural imaging studies have recently demonstrated reductions in functional activity within the cortical and subcortical regions responsible for convergence control in patients with both acquired and developmental CI.^39^^,^^40^

Reflexive fusional vergence also possesses robust short-term adaptive capacities that allows us to maintain efficient and precise binocular alignment throughout our lives.^41^^–^^46^ Experimentally, this plasticity can be studied by shifting the target stimulus before the eyes arrive at the intended location. Such stimuli are known as double-steps and were originally developed for the study of saccadic plasticity.^47^^,^^48^ In this paradigm, the second shift in target location creates a perceived error in the initial preprogrammed motor response. If an individual is consistently exposed to such a stimulus, the amplitude and peak velocity of subsequent saccades or vergence are altered in order to compensate with an aim to minimizing the experimentally induced end-point error. A strong correlation between this form of plasticity in reflexive convergence responses and the successful use of multifocal spectacle lenses was recently reported,^49^ suggesting that it plays a role in our ability to successfully adapt to changes in our visual environment.

We have reported directional asymmetries between reflexive fusional convergence and divergence responses in healthy, binocularly normal controls, which also extended into their adaptive capacities.^50^^,^^51^ In addition to being slower at baseline than convergence, reflexive divergence responses demonstrated limited recruitment of larger, faster responses after completion of an adaptive lengthening double-step paradigm.^52^ This result suggests that a saturation limit in the preprogramed pulse-generating divergence neural mechanism was reached. Beyond this limit, the width of the velocity profile increased in response to the double-step stimuli; however, the overall efficacy of this alterative process was significantly reduced.^52^

The following study aims to test two separate, but dependent, hypotheses in order to better characterize the oculomotor deficits that underpin the clinical condition of convergence insufficiency. First, individuals with convergence insufficiency will demonstrate a reduced capacity to adaptively lengthen their convergence responses when compared to binocularly normal controls. Second, this reduced adaptive plasticity will be associated with a sluggish or potentially saturated underlying reflexive (convergence) mechanism.

## Methods

### Participants

A total of 10 participants with CI and 10 age-matched binocularly normal control (BNC) participants were recruited from the undergraduate and graduate student population at the University of Waterloo. The research protocols were approved by the University of Waterloo institutional review board and adhered to the tenets of the Declaration of Helsinki. To be included in either study group, subjects were required to have monocular visual acuities greater than 6/7.5. Exclusion criteria for both groups included a history of previous ocular surgeries, major injuries, or diagnosed traumatic brain injuries. All screening tests were completed through the subject's habitual refractive correction. The clinical methods and results of the screening tests are detailed in the Table.

**Table.** Clinical Assessments of the Control and Convergence Insufficiency Groups

Control Group											
Participant	MSRE	Age (y)	Stereopsis (arcsec)	Facility (cpm)	Phoria (6 m)	Phoria (40 cm)	NPC (cm)	PFV (40 cm)	NFV (40 cm)	CISS Score	Sheard's Ratio
S1	–7.25	28	120	12	–2	–2	2	40	–16	2	19
S2	0	21	30	20	–0.5	–3	0	16	–14	7	4.3
S3	–0.5	27	60	16	1	2	2	20	–8	14	11
S4	–1.75	32	30	21	1	3	0	35	–18	0	12.7
S5	–1.5	22	120	18	–3	–5	0	35	–25	0	6
S6	–4.5	23	30	25	2	4	0	40	–14	2	11
S7	0	31	60	16	–4	–7	3	45	–25	16	5.4
S8	–4.5	23	60	15	0	–2	4	30	–14	10	14
S9	–1	22	30	17	0	–1	0	35	–20	3	34
S10	–5	30	60	14	–2	–4	0	40	–16	2	9
Mean (SD)	–2.6 (2.5)	25.9 (4.2)	60.0 (34.6)	16.1 (2.9)	–0.75 (1.9)	–1.5 (3.6)	1.1 (1.4)	33.6 (9.2)	–17.0 (5.2)	5.6 (5.9)	12.6 (8.7)
**Convergence Insufficiency Group**											
**Participant**	**MSRE**	**Age (y)**	**Stereopsis (arcsec)**	**Facility (cpm)**	**Phoria (6 m)**	**Phoria (40 cm)**	**NPC (cm)**	**PFV**	**NFV**	**CISS Score**	**Sheard's Ratio**
S11	–2	22	120	9	–3	–12	8	16	–18	22	0.33
S12	–0.75	27	60	5	0	–6	4	10	–18	26	0.25
S13	–1.75	26	240	9	–1	–8	9	14	–12	23	0.75
S14	–0.25	34	120	6 (s)	–1	–10	15 (s)	6 (s)	–12 (s)	20	0.2
S15	–3	21	60	8	0	–8	11	10	–14	23	0.25
S16	0	22	240	0 (s)	–4	–9	11 (s)	6 (s)	–10 (s)	30	0.33
S17	–6.25	24	60	5	0	–6	6	10	–14	27	0.67
S18	–1.75	34	30	12	0	–4	25	14	–18	6	0.5
S19	0	20	120	6	–1	–10	7	12 (s)	–14	28	0.2
S20	–1.75	24	60	12	0	–5	9	14	–12	22	0.17
Mean (SD)	–1.75 (1.9)	25.4 (4.7)	111.0 (74.9)	7.6 (3.7)	–1.2 (1.4)	–9.1 (1.9)	10.5 (5.9)	11.4 (3.2)	–15.2 (3.4)	23.8 (6.6)	0.36 (0.3)

Divergent (exo) values are negative and convergent (eso) values are positive. All phoria and fusional reserve values are expressed in prism diopters. The “(s)” denotes suppression in the absence of diplopia. Global stereopsis was assessed with the TNO random-dot stereoscopic vision test. Vergence facility was measured over the course of 60 seconds using the standard 3-base-in/12-base-out prism procedures at 40 cm while the participant views a single line of 0.2 logMAR vertical text.^54^ This target was the same used to measure PFV and NFV blur points (or break if no blur was reported) at 40 cm using a prism bar in free space.^55^ Heterophorias were measured using the alternating cover test at 6 meters and 40 cm.^56^ Sheard's ratio was defined as the difference between the near heterophoria and the compensating fusional vergence reserve, divided by the near heterophoria amplitude.^56^ NPC was measured using a single-letter 0.2 logMAR target that moved directly along the midline at a constant speed until the subject reported diplopia or the examiner observed one eye losing fixation and taking up an exotropic vergence posture. MSRE, mean sphere refractive error; PFV, positive fusional vergence; NFV, negative fusional vergence.

### Convergence Insufficiency Classification

Convergence insufficiency was defined primarily using the Convergence Insufficiency Treatment Trial criteria,^32^^,^^53^ where the main diagnostic criterion was a heterophoria exo-deviation that was at least 4 prism diopters greater at 40 cm than at 6 m.^2^^,^^32^ To meet the diagnosis of CI for this study, participants then also had to exhibit two or more of the following signs: (1) a receded NPC beyond 6 cm; (2) positive fusional reserves less than twice the amplitude of the near dissociated exophoria (failing Sheard's criterion, Sheard's ratio < 2); (3) a score ≥ 20 on the Convergence Insufficiency Symptom Survey (CISS); and (4) vergence facility below 13 cpm.^54^ Two CI participants (S17 and S19) had been previously diagnosed with CI and prescribed therapy; however, neither completed treatment and both remained symptomatic at the time of recruitment.

### Apparatus

Vergence responses were stimulated using two identical high-contrast crosses with suppression cues, presented dichoptically on two 7-inch liquid-crystal display monitors that were viewed at 40 cm in a mirrored haploscope. The subject's interpupillary distance was accounted for in the hardware to provide a congruent accommodative–vergence stimulus when the binocularly fused target was placed at the center of each monitor. This was the starting position of the stimulus for each disparity step change from which where vergence responses were measured. Head movements were limited by a custom chin and forehead restraint. Monocular eye movements were recorded at 250 Hz, digitized, and stored for offline analysis using the EyeLink II eye tracker (SR Research, Mississauga, Canada). A custom monocular nine-point calibration and validation procedure (48° horizontally and 16° vertically) was used at the beginning of each experiment and repeated anytime the participant sat back from the apparatus. A gaze error of less than 0.5° was required during validation testing at each point before continuing. The apparatus and visual stimulus parameters have been described in detail in other work.^52^^,^^57^

### Procedures and Stimuli

A complete description of experimental procedures with schematic illustrations has been described in our previous work^58^ and follows other investigations.^41^^,^^46^ Briefly, participants completed one screening visit to confirm their visual status (Table), followed by two separate experimental visits. One condition of vergence testing was completed per visit, with at least 5 days (maximum 15) between each visit. The order of the two experimental conditions was randomized and included convergence gain increasing (CGI) and divergence gain increasing (DGI). Each experimental trial contained a baseline, adaptation, and recovery phase and was confined to a single vergence direction (convergence or divergence). After completion of the baseline phase and again after the adaptation phase, the participant was given a break of up to 3 minutes before continuing.

The baseline and recovery phases were identical and consisted of 20 2° symmetric step changes in disparity (single-step) that were presented with a randomized delay to prevent prediction.^59^ The adaptation phase was comprised of 75 double-step stimuli and 10 2° single-step test stimuli (identical to that of the baseline and recovery phases). The double-step stimuli began with the same 2° disparity step, followed 175 ms later by an additional 1.5° step in the same direction. The test stimuli began only after 25 consecutive double-step stimuli had been presented and were randomly interleaved with the remaining 50 double-step stimuli at an average rate of 5:1.^46^

A 2° + 1.5° step amplitude was chosen for two reasons—the first was to ensure that the total angular convergence demand of the experimental stimulus did not exceed the maximum convergence limit of the individuals in the CI group (determined by their NPC) or the maximum fusional range of their disparity vergence system (determined by the break point of the fusional vergence reserves). This was necessary to ensure that any differences observed between the adaptive responses of the groups were not confounded by limitations in the oculomotor plant or disparity-driven vergence capacity in the CI group. The second reason was to limit the potential for saccadic interactions and maximize the number of vergence responses that were purely symmetric along the midline. This precaution is based on previous observations that larger step changes in disparity tend to generate responses containing significant conjugate (saccadic) components.^60^ These mixed, asymmetric vergence movements have disconjugate (vergence) peak velocities that are three to eight times greater when compared to responses that are purely disconjugate.^61^^–^^65^ Furthermore, in CI, they are encountered much more frequently and at lower step amplitudes.^66^

### Analysis and Statistics

Monocular eye positions were analyzed offline using a custom analysis package designed in MATLAB (MathWorks, Waltham, MA, USA).^57^ The difference between right- and left-eye positions defined the vergence angle, and a two-point central difference algorithm defined vergence velocity. Step vergence total response amplitudes were identified using a 1.5°/s start–stop velocity threshold. The settling time was defined by the difference between the time of movement onset and the time at which the vergence velocity was ≤0.5°/s for 32 ms consecutively. To mathematically isolate the initial, open-loop, reflexive vergence command of each response analyzed (henceforth known as the pulse response), a phase-plane analysis was employed. This type of analysis and its application to vergence responses have been summarized numerous times elsewhere.^45^^,^^57^^,^^67^ The [analysis] provides a means to estimate the amplitude of the open-loop pulse response if visual feedback was unavailable.

Baseline and recovery phase responses were binned sequentially into blocks of 10. In the adaptation phase, the 10 single-step test stimuli were binned separately and were used to define the adaptive changes in vergence response properties. Vergence responses containing saccades (conjugate response velocity > 40°/s) with latencies less than 80 ms were also excluded.^68^ The degree of adaptation within each subject's vergence system was defined as the percent difference between the mean of the last baseline bin response metric and corresponding measurement mean in the test response bin. Previous work has demonstrated that the majority of reflexive vergence adaptation under these circumstances occurs within the first 20 to 30 double-step stimuli.^46^^,^^57^

The datasets generally satisfied the assumption of normality (Shapiro–Wilk test, *P* < 0.05). Where deviations from normality were significant, non-parametric equivalent testing was used (Mann–Whitney *U* tests). Two-way ANOVAs were used to assess the effect of the test conditions (CGI vs. DGI) and group (control vs. CI) on the vergence response parameters measured in the baseline, adaptation, and recovery phases of the experiment. Bonferroni-corrected post hoc testing was then used to compare differences between groups on specific conditions.

## Results

### Clinical Screening Differences

Control and CI participants did not differ statistically in age (*t* = 0.9, *P* = 0.4), mean sphere refractive error (*t* = 0.1, *P* = 0.96), distance heterophoria (*t* = 0.59, *P* = 0.55), or negative fusional vergence (*t* = 1.7, *P* = 0.11). CI participants had significantly higher levels of exophoria at near (*t* = 5.4, *P* < 0.001), greater NPCs (*U* = 0.5, *P* < 0.001), and higher CISS symptom scores (*U* = 2.5, *P* < 0.001); they also had significantly lower vergence facility (*U* = 0.5, *P* < 0.001), positive fusional vergence (*U* = 7.3, *P* < 0.001), and Sheard's ratio (*U* = 0, *P* < 0.001). Global stereopsis was better in the control group, but the difference did not reach statistical significance (*U* = 27, *P* = 0.09) in part due to the greater variation in the CI group.

### Baseline Vergence Response Differences

**Figure 1.**
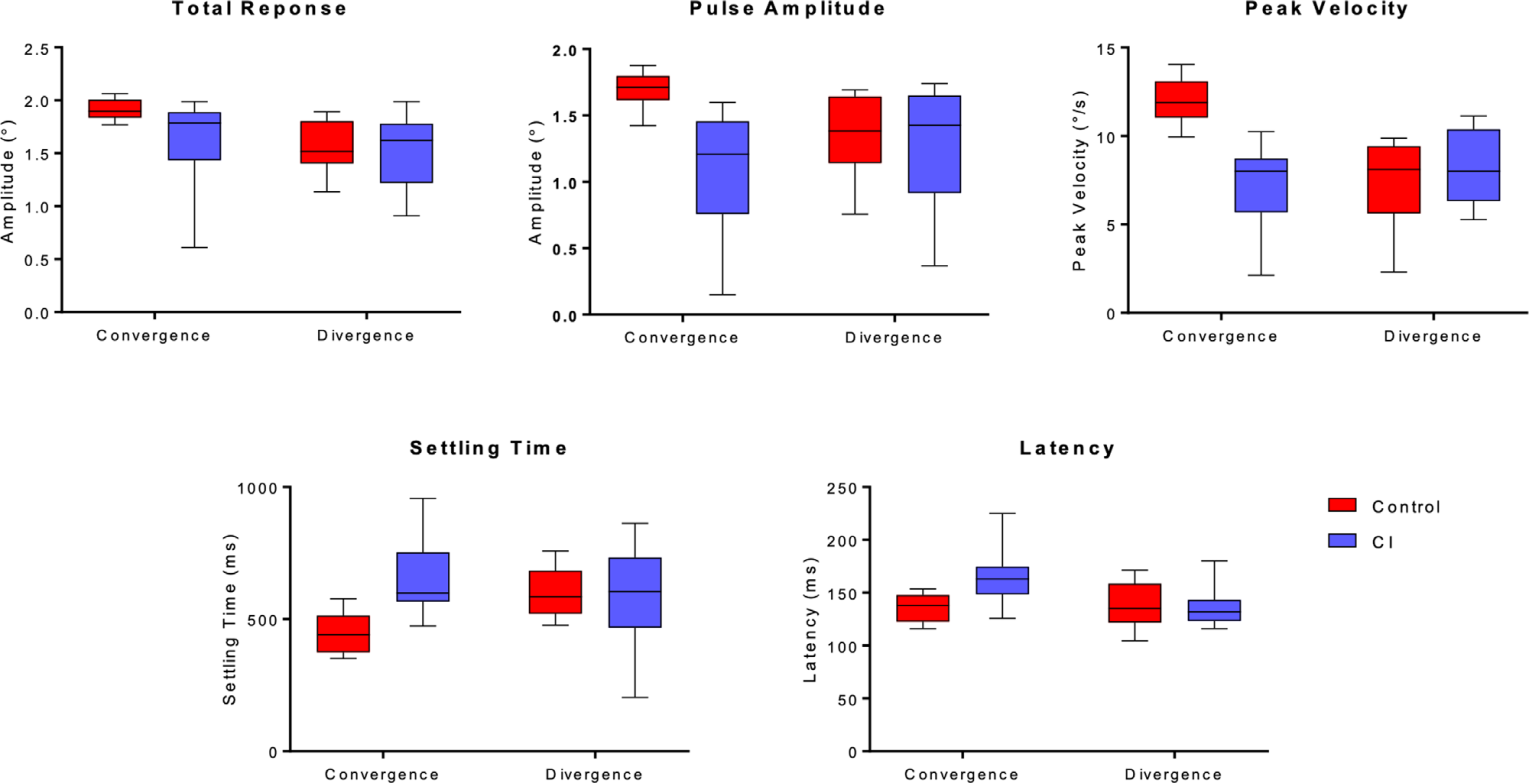
Properties of vergence responses to the 2° disparity step stimulus in the final block of the baseline phase. Box plots represent the within-group variance of the mean of each parameter.

**Figure 2.**
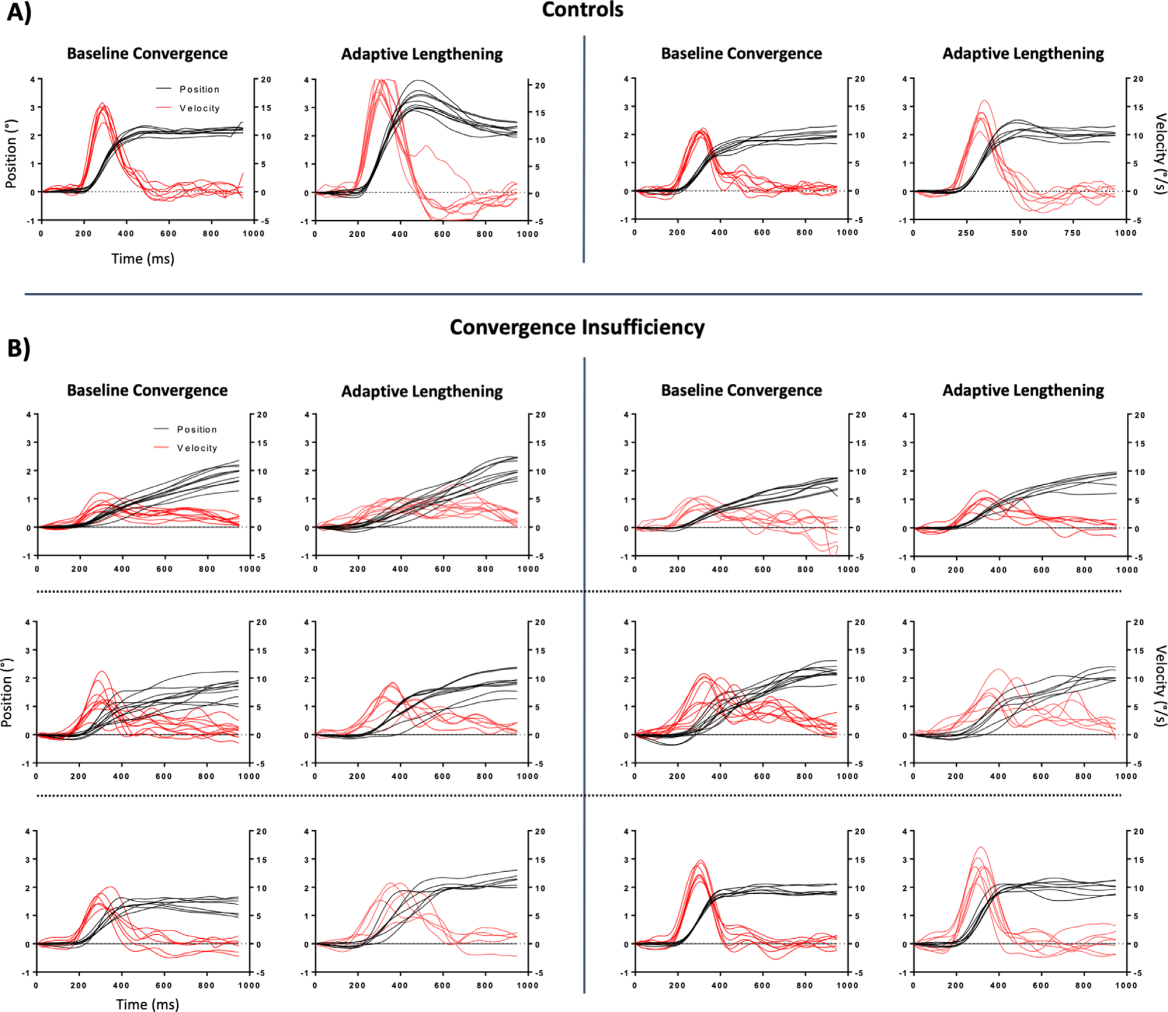
Convergence response amplitude (*black*) and velocity (*red*) traces for two BNC participants (**A**) and six CI participants (**B**). Baseline convergence plots are the last block of 10 responses in the baseline phase. Adaptive lengthening plots are the vergence responses to the test stimuli during the adaptation phase. The subject plots in the middle-left rows of (**B**) show an example of one of the CI participants who was unable to fuse the convergence step stimuli during the latter portions of the adaptation phase.

**Figure 3.**
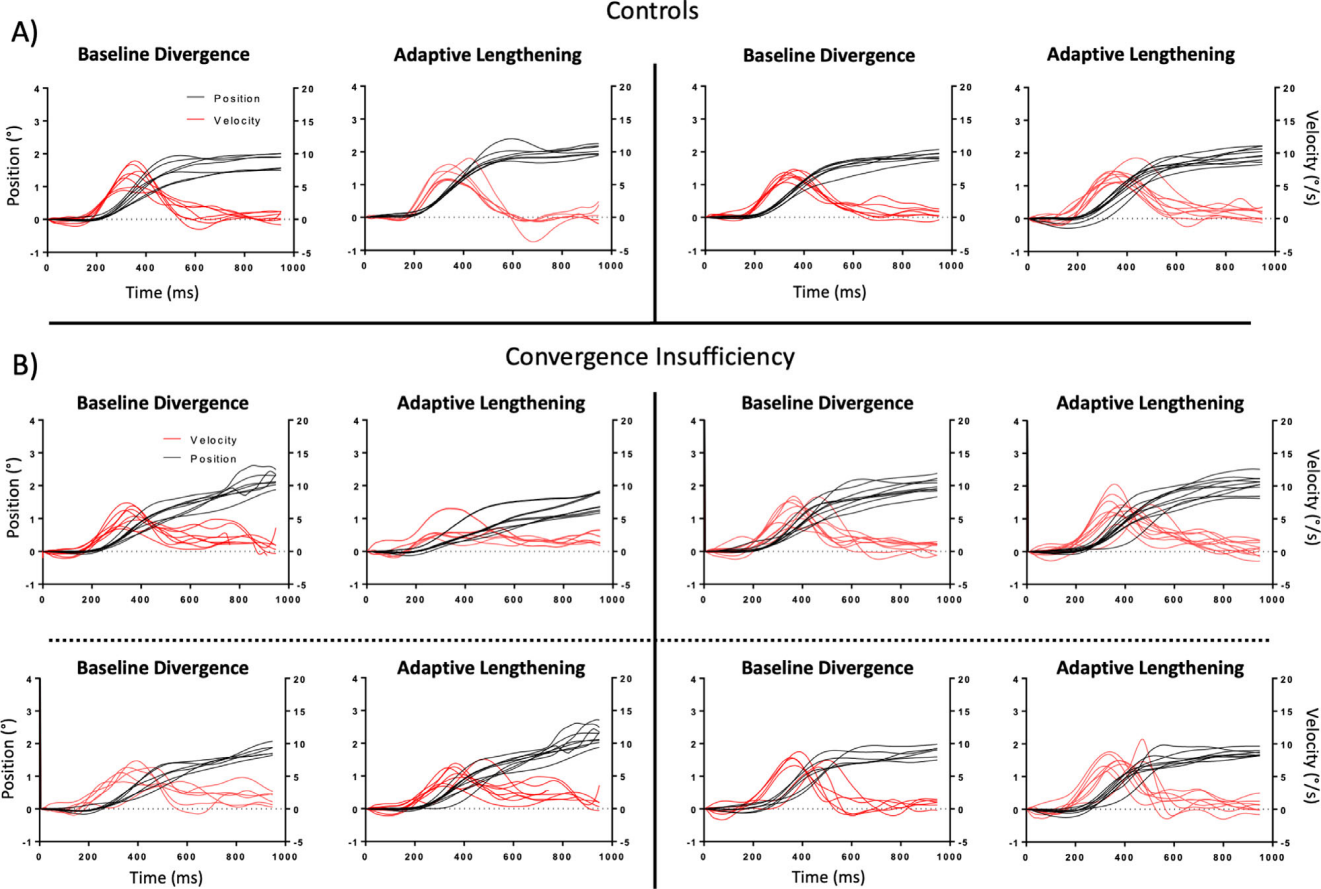
Divergence response amplitude (*black*) and velocity (*red*) traces for two BNC participants (**A**) and four CI participants (**B**). Baseline divergence plots are the saccade-free responses from the last block of the baseline phase. Adaptive lengthening plots are the vergence responses to the test stimuli during the adaptation phase. The subject plots at the top left in **B** show an example of one of the CI participants who was unable to fuse the divergence step stimuli during the latter portions of the adaptation phase.

As expected and consistent with previous work,^27^^,^^29^^,^^30^^,^^39^ there was a main effect of group in the baseline vergence pulse response amplitude [*F*(1, 43) = 5.7, *P* = 0.02], peak velocity [*F*(1, 43) = 13.5, *P* < 0.001], and settling time [*F*(1,*P*43) = 9.5, *P* = 0.004]. There was also a significant interaction between group and condition in these baseline parameters [pulse amplitude *F*(1, 43) = 3.9, *P* = 0.05; peak velocity *F*(1, 43) = 5.8, *P* = 0.02; settling time *F*(1, 43) = 2.7, *P* = 0.03]. The results of the baseline analysis are summarized with boxplots in Figure 1. The baseline panes in Figure 2 provide sample convergence traces from 1 BNC and 3 CI participants. The same panes in Figure 3 provide sample divergence traces from 1 BNC and 2 CI participants.

Post hoc tests indicated that the BNC group had significantly large preprogrammed (pulse) baseline convergence responses compared to the CI group (1.7° ± 0.2° vs. 1.1° ± 0.5°, *P* = 0.02; 12.0°/s ± 1.8°/s vs. 7.4°/s ± 2.5°/s, *P* < 0.001). No significant differences were found between the two group's baseline divergence response dynamics in this analysis (pulse amplitude: 1.4° ± 0.3° vs. 1.3° ± 0.6°, *P* = 0.98; peak velocity: 8.3°/s ± 2.1°/s vs. 7.5°/s ± 2.7°/s, *P* = 0.8). Interestingly, there were no significant differences between baseline convergence responses in the CI group and the baseline divergence responses in the BNC group (*P* > 0.5). The only convergence response parameter that did not have a significant effect of group was total response amplitude [*F*(1, 43) = 2.9, *P* = 0.67]. Finally, there was a significant effect of group in response latency [*F*(1, 43) = 6.8, *P* = 0.01]; however, there was no significant effect of condition [*F*(1, 43) = 0.2, *P* = 0.7], nor was there a significant interaction [*F*(1, 43) = 1.7, *P* = 0.21]. From Figure 1, it appears this effect could be related to the latency in the CI group baseline convergence responses.

The between-subject variability of the baseline convergence responses was clearly larger in the CI group (Fig. 1); however, there did not appear to be any systematic differences in the variability of divergence responses between the two groups. The mean number of movements analyzed from the baseline phase was not different between groups for a given direction (convergence: control 84.5% ± 9% vs. CI 76.2% ± 14%, *P* = 0.11; divergence: control 81.4% ± 7% vs. CI 73.8% ± 14%, *P* = 0.13)*.*

### Reflexive Vergence Adaptation Differences

**Figure 4.**
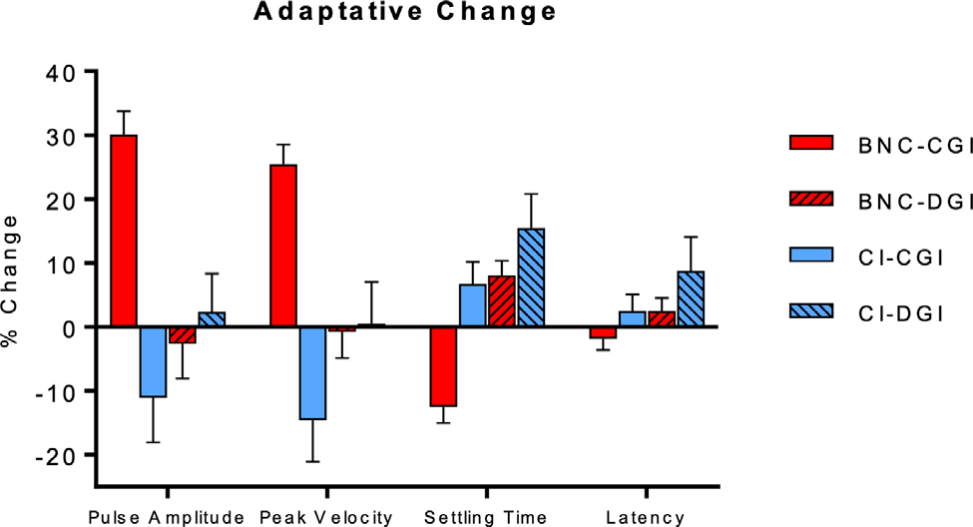
Mean (SD) of the normalized percent change in vergence response properties to the test stimuli in the adaptation phase compared to the last baseline block.

Figure 4 summarizes the normalized percent change of vergence response parameters to the test stimuli in the adaption phase compared to the baseline phase for both stimulus directions in each group. The same results for a sample of subjects are depicted graphically in Figures 2 and 3. There was a main effect of group in the change in vergence pulse amplitude [*F*(1, 34) = 6, *P* = 0.02), peak velocity [*F*(1, 34) = 13, *P* = 0.001], and settling time [*F*(1, 34) = 7, *P* = 0.01]. There was also an interaction effect between group and condition in the pulse amplitude [*F*(1, 34) = 22, *P* < 0.001] and peak velocity [*F*(1, 34) = 15, *P* < 0.001]. A main effect of condition was also significant for the settling time [*F*(1, 34) = 5, *P* = 0.04].

Post hoc tests showed that the adaptive increase of the convergence pulse response amplitude (30.2% ± 11.4% vs. –11.1% ± 22.1%; *P* < 0.001) and peak velocity (25.4% ± 9.8% vs. –14.6% ± 20.6%; *P* < 0.001) in the BNC group was significantly greater than what was observed in the convergence responses of the CI group. There was no significant change in the mean pulse amplitude [*t*(9) = 0.4, *P* = 0.7] or peak velocity [*t*(9) = 0.9, *P* = 0.38] after adaptive lengthening in the CI group. In terms of divergence adaptive lengthening, there was no difference between groups (pulse amplitude *P* = 0.43; peak velocity *P* = 0.99); however, the increases in convergence response amplitudes (*P* < 0.01) and peak velocities (*P* < 0.01) were greater in the control group than the divergence adaptive responses of either group. Of interest, the adaptive changes in the CI participants’ convergence responses were no different from their own divergence adaptive responses (*P* = 0.2) or the divergence adaptive response of the BNC group from either group (*P* = 0.3). The settling time of the BNC convergence responses was significantly reduced post-adaptation [*t*(9) = 5, *P* < 0.001]. This change in settling time was significantly different between the BNC and CI groups (*P* = 0.02), whereas there was no difference between groups in the change in the divergence response settling times (*P* = 0.9) or the convergence response settling time of the CI group (*P* = 0.63).

**Figure 5.**
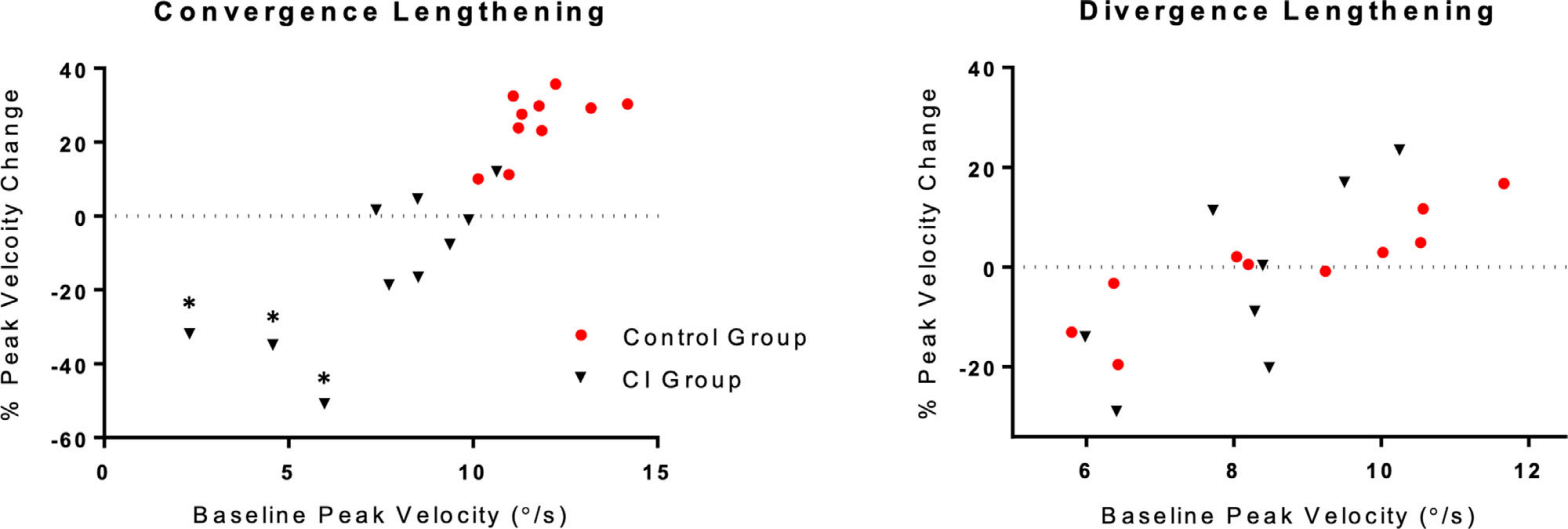
Scatterplots of the mean normalized percent peak velocity change after adaptation compared to the baseline mean for each participant. Asterisks in the left panel are placed above the CI participants’ data where large reductions in peak velocity were observed after adaptive lengthening of their convergence responses.

Qualitatively, there was a large degree of variation within the CI group's convergence adaptation data. To illustrate this point, Figure 5 plots the percent change in vergence pulse amplitude and peak velocity against the baseline value of the corresponding parameter. Three participants in the CI group demonstrated noticeable reductions in the dynamic properties of their convergence responses during and after adaptive lengthening. These three subjects also had the slowest baseline convergence responses and reported difficulty fusing the convergence disparity step stimuli (two of the three reported suppression before completion of the vergence facility test during screening) (Table). Interestingly, the CI participant with the fastest baseline convergence responses was also the only participant to show a significant increase in their convergence pulse response amplitude and peak velocity post-adaptation. This was the same participant who had the lowest CISS score. A similar relationship between baseline vergence dynamics and magnitude of adaptation was observed in the divergence condition in two of these three CI patients. An example of their convergence datasets is depicted in the top row of Figure 2. This was not observed in the remaining seven CI participants, where small increases in vergence response amplitude were accompanied by increases in response duration with no significant changes in the reflexive pulse response amplitude or peak velocity.

The participants with the slowest baseline reflexive responses also exhibited a decrease in the pulse response amplitude and peak velocity after adaptation (Fig. 5). To ensure that the data of these three CI subjects were not solely responsible for the between-group effects reported, their data were excluded and the between-group analysis described above was run again. The results of this re-analysis were still significantly different across the same parameters, stated earlier in this section (*P* < 0.02). Importantly, the overall change in the pulse response dynamics after adaptation was still not significantly different from zero in this subgroup of CI subjects (*P* > 0.15).

There was no significant change at the group level between conditions in the vergence response latency [t(9) > 1.6, *P* > 0.15]. The average number of convergence responses that were free of blinks or significant saccadic intrusions to the test stimuli in the adaptation phase differed significantly between groups (control: 82.2% ± 9%, CI: 59.5% ± 21%; *P* = 0.009). This was due to an increase in the number of convergence responses containing saccadic intrusions in the CI group during adaptation (12.7% ± 10% increase from baseline; *P* = 0.01). Control participants had significantly more divergence responses to the test stimuli that were free from saccades and blinks when compared to CIs (76.6% ± 9% vs. 61.4% ± 14%; *P* = 0.01); however, the number of responses excluded due to saccadic intrusions was not significantly increased from baseline in the CI group (7.4% ± 15%; *P* = 0.16).

### Recovery Post-Adaptation

**Figure 6.**
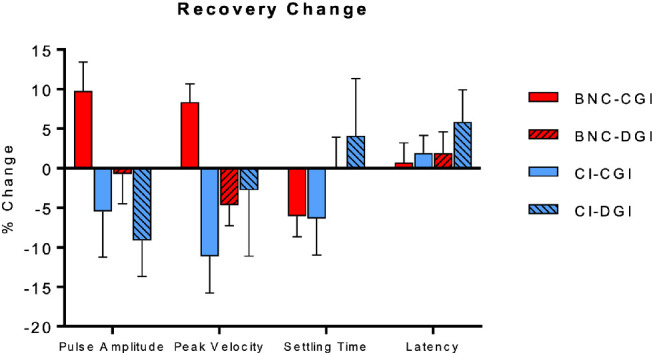
Mean (SD) of the normalized percent change in vergence response properties in the recovery phase compared to the last baseline block.

Figure 6 depicts the normalized group mean change in vergency response parameters between the final block (last 10 responses) of the recovery phase and the last baseline block. There was a main effect of group for both pulse amplitude [*F*(1, 34) = 6.8, *P* = 0.01] and peak velocity [*F*(1, 34) = 5.3, *P* = 0.03]. For both parameters, the mean convergence responses of the BNC group remained larger than at baseline (positive percentage change), whereas the remaining three group conditions exhibited no change or an overall reduction (negative percentage change) in these parameters. This was most notable in the CI group's convergence and divergence pulse response amplitudes and peak velocities. The mean negative change observed in Figure 6 reflects the participants who struggled to obtain fusion of the disparity step stimuli after the baseline phase.

## Discussion

The purpose of this study was to investigate the effects of convergence insufficiency on reflexive fusional vergence and its plasticity with an aim to elucidate a more direct understanding of the mechanisms underlying this disorder and the diagnostic value of different oculomotor parameters, specifically reflexive fusional vergence and its plasticity. Consistent with previous literature, our participants with CI demonstrated markedly reduced reflexive convergence response dynamics, accompanied by longer response durations and settling times. This observation supports the diagnostic value of these parameters with the appropriate instrumentation. In addition, attenuated fusional convergence response dynamics were associated with reduced adaptive capacities, illustrating a functional impairment of the reflexive fusional convergence mechanism, which is fundamental to the maintenance of bifoveal fixation, motor fusion, and stereopsis.

The novel and most significant result of this study was the impaired plasticity observed in the reflexive fusional convergence of the CI group, for which the adaptive lengthening stimuli failed to induce any significant increases in the subsequent response kinematics. Instead, an increased recruitment of saccadic influences in convergence responses was noted in the CI group. Interestingly, attenuated convergence plasticity in the CI group was also observed in their own divergence responses and those of the BNC group to the double-step stimuli. The negative change in the convergence response dynamics in the CI group that was observed in the recovery phase suggests that fatigue may have also occurred in a portion of this population. This is consistent with recent results that have characterized more rapid fatigue in the reflexive fusional convergence responses of patients suffering from traumatically induced CI.^31^

A second aim of this study was to test the hypothesis that attenuated reflexive vergence responses would be unable to recruit larger motor responses to increased stimulus demands, thus suggesting saturation of the reflexive fusional response mechanism. The similarities between the divergence data in each group and the convergence data in the CI group support this hypothesis. Overall, the participants with the smallest and slowest reflexive pulse fusional responses demonstrated the least degree of adaptive modulation, regardless of the group or disparity direction. The opposite was also true for participants with larger, faster initial baseline vergence response properties (Fig. 5).

An alternative hypothesis would be that the angular vergence demand of the experimental stimuli exceeded the limits of the vergence plant and/or the disparity-driven vergence mechanism and therefore would have prevented the recruitment of a larger, faster response to the double-step stimuli. The reduced vergence capacity exposed in the clinical screening metrics was not larger than the maximum demand of the experimental double-step stimuli, nor did the divergence demand exceed parallel gaze (0°); therefore, this alternative hypothesis can be ruled out. The directional effects in the BNC group also further rule out plant limitations as a confounder in our interpretation of the current results. Of additional interest to this discussion is that the CI group's preprogrammed pulse response behavior suggests saturation far before the plant or fusional vergence ranges would begin to impose physical limits. Therefore, we would predict that a similar attenuated adaptive response would be observed in the BNC group's convergence responses when the disparity demand of the double-step stimuli exceeded the maximum amplitude of the preprogrammed pulse responses. Understanding how this saturation limit may map to clinical metrics would be a valuable tool in the diagnosis and management of CI.

It is unclear whether the recruitment of more saccadic–vergence responses in the CI group was a separate adaptive mechanism in response to the double-step stimuli or was the result of central fatigue in the motor substrate. Others have shown these types of saccadic–vergence responses to be more frequent in CI.^66^^,^^69^ Their frequency reduces as symmetric vergence response dynamics increase with successful treatment.^66^ It would be interesting to explore the faciliatory effects of such saccadic–vergence interactions on adaptive behavior in future work; however, they would be difficult to compare with controls, given their relative scarcity in our current dataset.

We found no significant differences in baseline reflexive fusional divergence parameters between study groups, consistent with previous literature.^39^^,^^70^ These findings suggest that, although the CI group's heterophoria was more exophoric at near (and this was not compensated for in our dichoptic apparatus), this did not facilitate the divergence response dynamics or its their degree of plasticity. Taken together, these findings confirm that convergence and divergence are truly separate neural substrates^67^^,^^71^^,^^72^ and that a motor impairment in one mechanism does not impair or facilitate the development or response mechanics of the opposing vergence substrate.

Cell recordings in primate vergence motor and premotor areas have indicated that vergence response amplitudes to step changes in retinal disparity are well correlated with the duration of neuronal firing and that vergence velocity is well correlated with the neuronal firing rates.^71^^–^^73^ Based on these neurophysiological data, our results can be interrupted to suggest that limited increases in the peak velocity of reflexive vergence responses after adaptive lengthening adaptation are demonstrative of a ceiling effect in the neural firing and/or recruitment rates. Neural imaging data from participants with CI have also shown an overall reduction in the functional activity of the cortical and subcortical vergence regions when compared to healthy controls.^39^^,^^40^ Thus, the current evidence suggests that CI is underpinned by an underdeveloped or impaired reflexive (disparity-driven) fusional convergence mechanism. This results in an impairment in the overall efficacy of adaptation. By extension, then, therapies that employ techniques such as Brock string training, convergence cards with rapid fixation changes, or prism-based vergence facility training that target the disparity-driven preprogrammed reflexive fusional response should result in the most efficacious initial improvements in signs and symptoms of CI. This assumes that this system is amenable to rehabilitation, which there is significant evidence to support.^29^^,^^74^ In moderate to severe cases of CI, where fusion is still obtainable but suspectable to breakdown, therapies that target other impediments to binocular fusion, such as disparity sensitivity (stereoacuity), may be necessary to facilitate full rehabilitation. These results suggest that objective measurements of reflexive fusional vergence dynamics could also serve as biomarkers of CI and its resolution through treatment.

An unexpected finding in the baseline vergence data was the longer reflexive convergence response latencies in the CI group. There is a paucity of data providing similar comparisons between CI and control group convergence response latencies in the literature. The studies that provide the most detailed analysis of convergence response dynamics either fail to report response latencies^27^^,^^39^^,^^66^^,^^70^ or do not compare the findings between groups.^29^^,^^30^ One study did find differences between traumatically induced convergence latencies of the CI and control groups.^7^ The authors of this study also reported greater divergence latencies in their traumatic CI group than controls. This was not the case in our results. The latency differences reported in the previous works cited are likely more representative of the different etiologies of CI (traumatic vs. *presumed* developmental) or experimental platform differences. It is possible that the greater reflexive convergence response latencies in our CI group represent an upstream sensory processing issue with retinal disparity. The larger and more variable global random-dot stereoacuity thresholds in our CI population could be taken to support such a conclusion. Others have not reported differences between groups on these stereoacuity parameters,^75^ and our limited sample size may reduce the external validity of the stereoacuity findings reported. Future work in such patient groups should focus on characterizing stereoacuity thresholds in greater detail with more rigorous psychophysical protocols to provide a greater understanding of the sensory status of such populations.

The capacity to adaptively lengthen convergence responses has been associated with successful adaptation to progressive, multifocal lens wear in emerging presbyopes.^49^ Intuitively, these two functions (vergence plasticity and successful spectacle adaptation) could be related, as multifocal lenses induce asymmetric geometric distortions of the retinal images. This altered sensory input requires rapid recalibrations of binocular alignment and other (oculo)motor control systems, such as the vestibular–ocular response gain, in order to maintain optimal performance and visual comfort. Failure to properly adapt these responses, such as observed in the reflexive vergence of our small sample of CI subjects, could then contribute to a poor experience and increased rates of maladaptation. Taking this into account, the clinician should be mindful of the patient's binocular function when prescribing multifocal lenses or when altering the magnitude of prescribed spectacle anisometropia in patients with reduced convergence function.

## Conclusions

The generalizability of these results and the conclusion of this study would be validated by additional data characterizing the effects of convergence therapy on these adaptive responses in CI. We would expect that improved reflexive convergence response dynamics during and after therapy would underlie an improvement in adaptive capacities. Others have provided evidence that this would be the case^42^; however, their study used the same error-based disparity double-step paradigm as both the rehabilitation therapy and the outcome measure. As a result, task-specific explicit learning mechanisms could not be ruled out as a confound.

This study was designed to characterize the adaptive capacities of reflexive vergence in patients with convergence insufficiency and contrast it with that of binocularly normal controls. The results confirm that reflexive convergence responses to step changes in retinal disparity are significantly reduced in CI. Importantly, these individuals also exhibited a limited capacity to adaptively lengthen their reflexive convergence responses through the recruitment of faster reflexive responses, as is the case in controls. Reflexive divergence adaptive responses were less robust when compared to convergence in controls and found to be similar between groups. These results add to the growing body of behavioral and neural imaging data suggesting that convergence insufficiency is the result of a generally reduced or impaired reflexive convergence neural substrate. To the best of our knowledge, the data provide the first assessment of short-term sensorimotor adaption of vergence in convergence insufficiency and provide new insights into the functional oculomotor deficits in these patient populations and the neurophysiological underpinnings of convergence insufficiency.
